# Liquid Biopsies in Follicular Thyroid Carcinomas—A Brief Report

**DOI:** 10.3390/diagnostics14141577

**Published:** 2024-07-22

**Authors:** Marie-Louise Uhre Hansen, Simone Kloch Bendtsen, Kathrine Kronberg Jakobsen, Ane Yde Schmidt, Christoffer Holst Hahn, Christian von Buchwald, Christian Grønhøj

**Affiliations:** 1 Department of Otorhinolaryngology, Head and Neck Surgery and Audiology, Copenhagen University Hospital, Rigshospitalet, 2100 Copenhagen, Denmark; 2 Center for Genomic Medicine, Copenhagen University Hospital, Rigshospitalet, 2100 Copenhagen, Denmark; 3 Department of Clinical Medicine, Faculty of Health and Clinical Sciences, University of Copenhagen, 2200 Copenhagen, Denmark

**Keywords:** follicular thyroid cancer, liquid biopsies, cell-free DNA

## Abstract

Thyroid cancer (TC) represents a significant health burden globally, with follicular thyroid cancer (FTC) posing diagnostic challenges despite advancements. This pilot study aimed to evaluate the utility of a liquid biopsy with cell-free DNA (cfDNA) in patients with FTC. Blood samples were collected from 13 patients diagnosed with FTC, DNA extraction was performed, and cfDNA was analyzed using the Illumina’s TruSight Oncology 500 High-Throughput panel. The results revealed low tumor mutational burden and minimal pathogenic variants in cfDNA, indicating challenges such as low DNA yield and poor material quality despite adequate coverage. Our findings indicate that cfDNA as an add-on diagnostic tool in patients with FTC might not be a useful supplement.

## 1. Introduction

Thyroid cancer (TC) is the most prevalent endocrine cancer worldwide, with an increasing incidence but decreasing mortality rates [1,2,3,4]. This form of cancer presents with a range of subtypes and is commonly diagnosed among individuals in their younger years. Among these, follicular thyroid cancer (FTC) constitutes approximately 10–18% of all thyroid cancer cases, making it the second most common subtype following papillary thyroid cancer (PTC) [1,5]. FTC tends to exhibit greater aggressiveness, often metastasizing to the lungs and bones, while PTC more commonly metastasizes to the lymph nodes [5,6]. Thyroid nodules are frequently encountered in clinical practice; however, accurately distinguishing between malignant and benign nodules continues to pose a significant challenge for healthcare professionals [7,8]. Despite extensive comprehensive diagnostic evaluations, including imaging studies and clinical tests, many patients undergo surgery to differentiate FTC from follicular neoplasia. Fine-needle aspiration biopsy is part of the diagnostic process, and it is insufficient for preoperative diagnosis because it is morphologically impossible to distinguish FTC from benign follicular thyroid adenoma. In some studies, follicular neoplasia carries a malignancy risk of 20–30% and up to 50% [9,10,11]. Consequently, a diagnostic hemithyroidectomy is necessary to secure a definitive histopathological diagnosis. Histopathology is also essential for further treatment regimens (completion of thyroidectomy, radioiodine therapy).

No molecular alterations have been widely implemented in FTC’s diagnostic, predictive, and prognostic workup. This gap underscores the need for reliable biomarkers to improve patient management. Biomarkers like thyroglobulin (Tg) and thyroglobulin antibodies (TgAb) are available for disease monitoring in specific thyroid cancer subtypes, but their effectiveness is limited. TgAb is detected in 23–29% of differentiated thyroid cancer cases, complicating disease recurrence monitoring, and preoperative Tg levels may be negative in 12% of cases, posing further challenges [12,13,14]. Calcitonin and carcinoembryonic antigen (CEA) serve as biomarkers in medullary thyroid cancer (MTC); however, calcitonin secretion may be absent in some MTCs, and CEA lacks specificity for MTC [14,15].

A novel, non-invasive cancer diagnosis and monitoring approach involve liquid biopsies, capitalizing on releasing intracellular material into the bloodstream during cellular processes like apoptosis and necrosis, particularly in cancer cells. Cell-free DNA (cfDNA), comprising DNA fragments released into the bloodstream, is detectable even in healthy individuals [16]. Elevated cfDNA levels are noticeable in certain benign, inflammatory, or precancerous conditions and individuals with cancer. Within cancer patients, a subset of cfDNA, known as circulating tumor DNA (ctDNA), harbors tumor-specific somatic mutations. Changes in the levels and molecular profiles of these circulating biomarkers reflect tumor dynamics, facilitating timely adjustments to therapy and personalized treatment strategies. Numerous studies have already indicated the promising potential of biomarkers for various other types of cancer. The use of cfDNA as a potential diagnostic tool regarding TC is debated and needs further investigation [17,18,19,20]. Much research has concentrated on PTC. In this pilot study, we aim to quantify cfDNA in blood samples and explore the potential of a prospective diagnostic biomarker in patients with FTC. Since FTC is a less common subtype and often exhibits different clinical behavior and molecular characteristics, focusing on FTC will address the existing gap in research. This study aims to provide valuable insights, potentially leading to improved diagnostic and treatment strategies for FTC, which remains underexplored compared to PTC.

## 2. Materials and Methods

This study was a prospective, observational clinical trial study. The study was approved by the Danish Data Protection Agency and the Regional Committee on Health Research Ethics (protecol number: H-17001863).

All patients (18 years or over) referred to the Department of Otorhinolaryngology, Head and Neck Surgery & Audiology, Copenhagen University Hospital, Rigshospitalet, with suspicion of head and neck cancer, were included in the study. The inclusion period was from 1 September 2020 to 1 December 2022. Blood samples were collected before a clinical diagnosis was established.

Patients who had a prior cancer diagnosis within the last three years were excluded, along with patients who were treated with chemotherapy and had a severe hematologic or immunological disease. Patients who had received any form of immune-suppressed medication within three years were also excluded. 

The European Thyroid Imaging and Reporting Data System (EU-TIRADS) was utilized to assess the risk of malignancy in thyroid nodules. This system is based on ultrasonographic evaluations and provides a standardized approach to categorize the likelihood of cancer in thyroid nodules.

### 2.1. Blood Samples and DNA Extraction

Two blood samples (2 × 10 mL) were collected from each patient using Streck cell-free DNABCT CE tubes (Streck Inc., La Vista, NE, USA) (stabilizes cell-free DNA). The collected tubes were stored at room temperature for up to three days before plasma isolation. The blood was then centrifugated at 2250× *g* for 10 min. After removing the buffy coat, the plasma underwent a second centrifugation at 16,800× *g* for 10 min and was transferred to a new tube, yielding approximately 4.5 mL of plasma per 10 mL of whole blood. The plasma samples were then stored at −80 °C until DNA purification and extraction. Cell-free DNA (cfDNA) was extracted using the Qiagen QiAMP ccfDNA minElute midi kit (Qiagen, Hilden, Germany) on a QiaSymphony, following the manufacturer’s instructions.

The libraries were prepared for tumor profiling with Illumina’s TruSight Oncology 500 High-Throughput (TSO500 HT, Illumina Inc., San Diego, CA, USA), a targeted next-generation sequencing (NGS) panel containing 523 cancer-related genes. It can identify relevant DNA variants of different tumor types and measure immuno-oncology biomarkers, microsatellite instability (MSI), and tumor mutational burden (TMB) [21]. The TSO500 protocol was performed according to the manufacturer’s instructions (except for omitting the first shearing step), and 20–40 ng of cfDNA was used as the input. The finished libraries were sequenced on Illumina’s NovaSeq 6000 (2 × 150 base pairs) with an average coverage of ×730 (range 269.6–1086.3). The raw sequencing data (.bcl files) were converted into FastQ files using Illumina’s bcl2fastq v2.20.0.

Sequenced reads were trimmed with BBduk v38.26 and mapped to the hg38/GRCg38 reference genome using BWA-MEM v0.7.15. Alignment quality control was performed with mosdepth v0.2.6. Somatic variants were called for each tumor sample with GATK v4.1.9.0 suite’s Mutect2 using Best Practises guidelines for tumor-only analysis (i.e., without a paired normal sample) and an internal “Panel of Normals” comprising cfDNA from “healthy controls” having been through initial health examinations with the suspicion of cancer but deemed cancer-free by expert medical personnel and pathologists. Copy number alterations were called using the local TSO500 app v.2.2.0 and TMB estimation.

### 2.2. Variant Filtering

Filtering of variants was performed using Qiagen’s online Clinical Insights (QCI) software vision 9.2.0.20230922. (Qiagen Bioinformatics Aarhus, Denmark/Redwood City, CA, USA). Variants more common than 0.5% in the 1000 genomes project, ExAC, genomAD, or NHLBI ESP exome were excluded. The final calls were made following a manual inspection of raw sequencing reads in the Broad Institute’s Integrative Genomic’s Viewer (V 2.13.1) [22].

## 3. Results

### 3.1. The Participants

**Figure 1:**
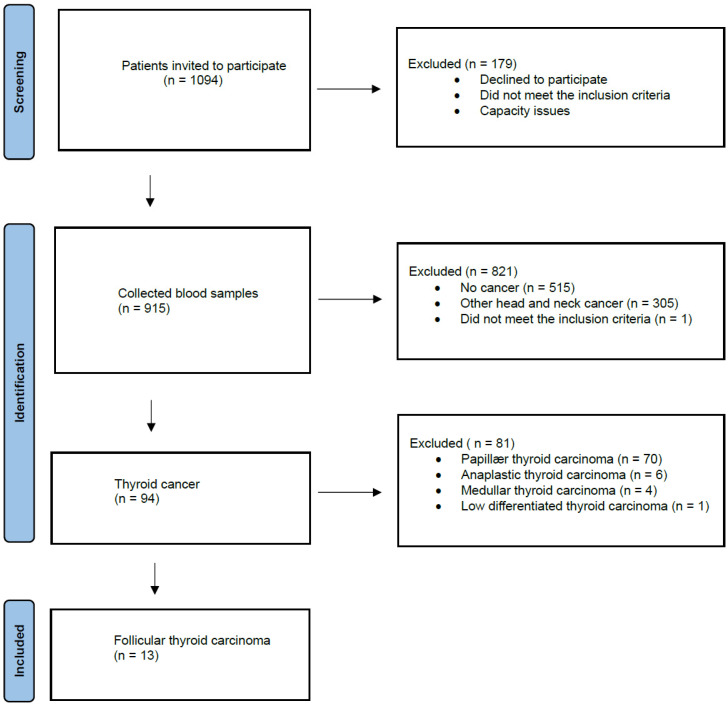
Flow diagram of study.

One thousand seventy-eight participants were invited to our study between 1 September 2020 and 1 December 2022. Nine hundred fifteen blood samples were collected from patients with suspicions of head and neck cancer, of which 94 were diagnosed with TC. Of these 94 TC patients, 13 were diagnosed with FTC and included in our study (Figure 1). The majority were female (61.5%, *n* = 8), and the mean age of diagnosis was 64 years (age range, 49–88 years) (Table 1). Two of the thirteen patients had a previous surgical history (2005, 2012) with hemithyroidectomy, where the pathology was benign. However, after thoroughly revising the pathology, the diagnosis was reinterpreted as a local relapse of FTC. The findings from the fine-needle aspiration demonstrated that follicular neoplasia was present in 46.6% of the FTC cases analyzed. The majority of patients had a primary tumor status (T) T2 (5 patients), region lymph node status (N) N0 (11 patients), and distant metastasis (M) M0 (10 patients) (Table 1).

### 3.2. DNA Results 

**Figure 2:**
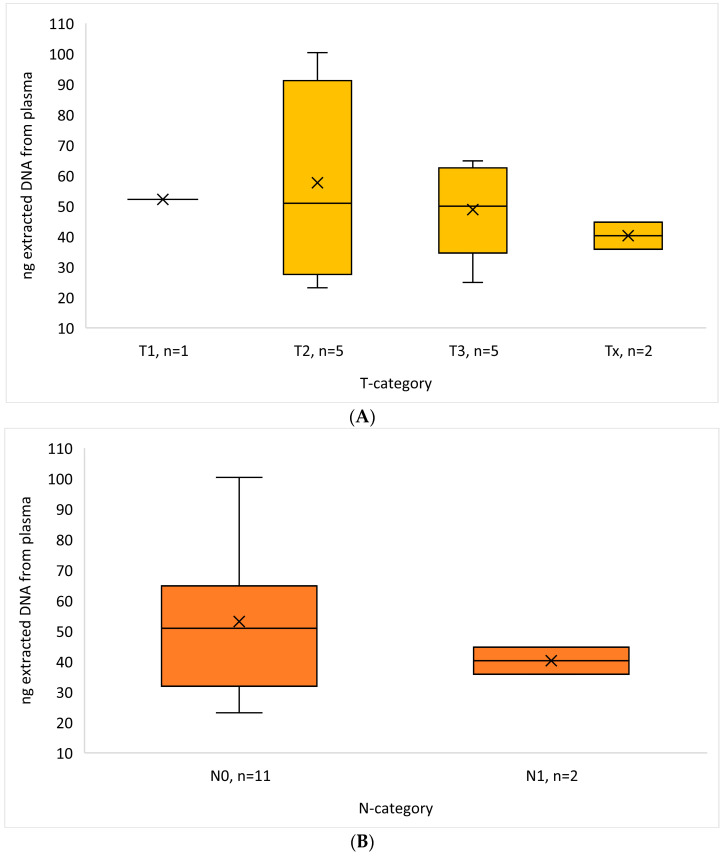
Box plot of extracted DNA from plasma (ng) in relation to (**A**) T-category and (**B**) N-category. Abbreviation: T-category, primary tumor status; N-category, region lymph node status.

We extracted a mean amount of 51.17 ng DNA from the samples (range 23.17–100.2 ng), and the amount did not correlate with the tumor category or lymph node category (Figure 2). All samples were found to have an overall low TMB status (range 0–2.4 mut/Mb) and a low amount of MSI (range 0–6). No variants classified as pathogenic or likely pathogenic were found in Table 2, and a few variants of unknown significance (VUSs) were found in Table 3. The five VUS mutations identified were in different patients and had few reads (5–16) even though the read depth was >500×. The identified variants were located on different chromosomes, and their impact was classified as missense mutations. Despite this, three out of the five variants were predicted to retain normal function, while the remaining two variants were predicted to result in a loss of function.

## 4. Discussion

In this pilot study, we examined the detection of cfDNA in 13 patients diagnosed with FTC. We found only a minimal number of pathogenic variants in the cfDNA (Table 2). Only in five of the thirteen patients were we able to identify VUS mutations where variants were on different chromosomes (Table 3). Furthermore, based on the existing literature, we were able to confirm that a cytologic diagnosis of follicular neoplasia obtained through fine-needle aspiration is insufficient to definitively rule out FTC [10,11] (Table 1). Several studies have shown promising results in detecting mutations in different TC subtypes (mostly PTC) and with different methods [19]. Genetic alterations and mutations are seen in over 90% of TC cases, particularly in PTC [23]. In PTC, *BRAF* fusions and *RET* fusions are frequently observed [18,24,25]. Genetic mutations in *BRAF* V600E are linked to higher disease-specific mortality, lymph node metastasis, extrathyroidal extension, an elevated risk of recurrence, distant metastasis, advanced disease stages, and older patient age [18,23,24]. The Catalogue of Somatic Mutations in Cancer (COSMIC) [26] reports the top 20 somatic mutations observed in FTC. The TSO500 contains 19 of these 20 genes [21]. The most common point mutations in FTC are in the *RAS* gene family or by *PAX8-PPARγ* rearrangements, and more are being investigated and associated with FTC [18,24,25]. *RAS* mutations are present in about 40% of FTCs and 20–40% of benign follicular adenomas [24]. Mutations in the *RAS* gene within follicular carcinomas are more significantly associated with increased tumor severity, a higher incidence of bone metastases, and an increased mortality rate [18,24]. In a systematic review from Zeyghami et al. [19], nineteen studies found the presence of mutated ctDNA in TC patients. For instance, the study by Zane et al. [20] identified *BRAF* V600E in 67.6% of thyroid tumor tissue samples but was not able to detect the mutation in plasma. They also discovered that cfDNA levels were elevated in patients with thyroid cancer compared to control individuals. Another study by Chuang et al. [27] analyzed 28 matched tumor and serum samples from patients with a range of thyroid disorders, including benign and malignant conditions. The researchers identified the *BRAF* V600E mutation in 35.7% of the cases with papillary thyroid tumor tissue samples. Additionally, they found that 21.4% of the cases also tested positive for the mutation in the serum samples. Patients with FTC, benign thyroid disease, or thyroid lymphoma were not able to detect *BRAF* V600E mutations in either tissue or blood samples. Pupilli et al. [28] measured the presence of *BRAF* V600E mutations in cfDNA across various groups. They found *BRAF* V600E mutations in 1.7% of healthy subjects, 6.4% of patients with non-nodular thyroid disease, 8.9% of those with benign thyroid nodules, 18.7% of patients with follicular lesions, and 27.1% of patients either suspicious for or diagnosed with malignancy. In 19 patients with PTC, *BRAF* V600E levels in cfDNA dropped significantly from 43.2% to 6.5% post-surgery. The mutation was more frequently detected in classical papillary carcinoma (72%) than in the follicular variant (54%). ROC curve analysis indicated that a *BRAF* V600E cfDNA level of 2.65% could distinguish patients with suspicious or diagnostic fine-needle aspiration results from healthy subjects, with 65% sensitivity and 80% specificity. This suggests that liquid biopsies could complement tissue-based biomarkers, offering a more comprehensive view of tumor dynamics and potentially detecting mutations missed in tissue biopsies. A study by Paulsson et al. [29] using whole-genome sequencing of FTC identified multiple mutations in established TC genes, including *TERT*, *NRAS*, *HRAS*, *KRAS*, *AKT*, *PTEN*, *PIK3CA*, *MUTYH*, *TSHR*, and *MEN1*. Recurrent somatic mutations were observed, with *FAM72D* and *TP53* mutations found in three cases each and *EIF1AX* mutations in three cases. Notably, *DGCR8* p.E518K missense mutations, which disrupt microRNA processing and are linked to familial multinodular goiter, were found in two cases. These mutations were associated with reduced *DGCR8* mRNA levels and a distinct miRNA profile, suggesting their role in thyroid carcinogenesis. 

There are several technical limitations in using liquid biopsies, such as low DNA yield, sequencing artifacts, and contamination, which may compromise the accuracy and reliability of results. One limitation of this study is the small sample size of only 13 patients, which hinders the generalizability of the results. During the filtering process in QCI, we detected several long indels, suggesting that the extracted DNA material was of notably poor quality despite the majority of samples having acceptable coverage. Approximately half of the cfDNA tests yielded negative results for the presence of any genetic variants. Even though the coverage appeared to be adequate, the quantity of ctDNA was insufficient (Table 2 and Table 3). Among the five samples tested, one sample had a coding mutation in *SDHA* (Table 2); this is often a pseudogene mutation, and one should be extra observant in low frequencies [30]. The presence of a pseudogene may obstruct the detection of sequence variations [30]. Only one exhibited a coding mutation (*RET*) in the top 20 FTC-related mutated genes [26] (Table 2). This observation underscores the challenges associated with detecting cfDNA in early-stage cancer patients, as the sensitivity of cfDNA measurement can be reduced due to the limited occurrence of recurrent mutations. This limitation seems relevant to our study since only two patients had distant metastasis (M1 stage), and none had T4a or T4b cancer (Table 1). Moreover, the poor quality of the DNA material and the insufficient quantity of ctDNA highlight the difficulties in relying on cfDNA tests for accurate variant detection in early-stage FTC. All samples were found to have an overall low TMB status, which is probably underestimated. The presence of long indels further complicates the analysis, potentially leading to false negatives or missed mutations. The negative results for variants in about half of the cfDNA tests suggest that the method may not be robust enough for early-stage FTC detection. At our institution, only variants with >10 reads are accepted diagnostically. False-positive and false-negative results may lead to diagnostic inaccuracies and inappropriate clinical decisions, highlighting the need for improved assay sensitivity and validation in diverse patient populations. The poor DNA quality and small sample size could be technical issues rather than definitive negative results, especially when combined with a low malignant cancer type.

We used the sequencing technology NGS and TSO500 platform, which allows for the detection of molecular alterations, including mutation copy number variations, providing insights into tumor biology, and guiding targeted therapy selection in many genes (523). NGS is a newer and more frequently used technology because it is cheaper and faster than traditional Sanger sequencing [21]. In contrast, the polymerase chain reaction method can only examine one or a few known gene mutations, which is not ideal in cases like here, where we do not know the specific gene profile. Additionally, the serial monitoring of ctDNA offers a real-time assessment of treatment response, disease progression, and the emergence of resistance mechanisms, facilitating timely adjustments to therapy and improving patient outcomes. Continued technological advancements in NGS platforms (a new TSO500 ctDNA v2 kit has been introduced to the market, aimed at enhancing sensitivity with reduced cfDNA input, achieving faster turnaround times, and offering a more streamlined workflow [21]), such as enhanced sensitivity, multiplexing capabilities, and streamlined workflows, will help improve the accuracy and reliability of liquid biopsy assays. 

## 5. Conclusions

In conclusion, while cfDNA is promising in the diagnosis of TC cancer, its widespread clinical use is hampered by methodological and validation limitations. 

Our study specifically investigated the application of liquid biopsy techniques in a small cohort of 13 patients with FTC. Unfortunately, we found that the liquid biopsy method, in its current form, proved to be unsuitable for this cohort. The inability to obtain reliable and sufficient results underscores the need to refine the technique further and the need for testing in larger cohorts. Achieving standardization, validating in larger cohorts, and establishing consensus on relevant markers are important. Our study also highlighted a practical limitation regarding the volume of material available for analysis. Specifically, we found that two Streck DNA tubes did not provide enough material to perform the assay sufficiently. This finding could suggest that either larger volumes of blood or more efficient methods for DNA extraction and analysis are needed to ensure adequate material is available for testing. A tissue sample is still needed to determine the correct histopathology of thyroid nodules.

## Figures and Tables

**Table 1:** Baseline characteristics of the patients.

	FTC + *n* (%)
**Sex**	
Female	8 (61.5)
Male	5 (38.5)
**Age, years**	
<45	0 (0)
45–50	2 (15.4)
50–60	4 (30.8)
60–70	2 (15.4)
70–80	4 (30.8)
80–90	1 (7.7)
CCI at diagnosis (not including the TC) *	
0	2 (15.4)
1	3 (23.1)
2	2 (15.4)
3	3 (23.1)
4	3 (23.1)
5	0 (0)
**Ultrasonography EU-TIRADS score**	
1	4 (30.8) **
2	0 (0)
3	4 (30.8)
4	4 (30.8)
5	1 (7.7)
**Fine-needle aspiration diagnosis**	
Follicular neoplasia	6 (46.2)
Malignant cells	2 (15.4)
Follicular carcinoma	1 (7.7)
To sparse material	1 (7.7)
No malignant cells	1 (7.7)
No fine-needle aspiration	1 (7.7) ***
Papillary thyroid carcinoma	1 (7.7)
**T-category**	
x	2 (15.4)
0	0 (0)
1a	0 (0)
1b	1 (7.7)
2	5 (38.5)
3a	4 (30.8)
3b	1 (7.7)
4a + 4b	0 (0)
**N-category**	
0	11 (84.6)
1a	0 (0)
1b	2 (15.4)
**M-category**	
x	1 (7.7)
0	10 (76.9)
1	2 (15.4)

Abbreviation: FTC, follicular thyroid carcinoma; CCI, Charlson Comorbidity Index; Eu-Triads, European Thyroid Imaging and Reporting Data System; T-category, primary tumor status; N-category, region lymph node status; M-category, distant metastasis. * The patients had the following comorbidities: reflux, hypertension, apoplexy, depression, and diabetes. ** Two of the four patients had a normal thyroid but a cystic metastasis on the neck. *** No FNA, hemithyroidectomy because of a huge left thyroid with a displacement of the trachea, EU-triads 1 (normal ultrasonic).

**Table 2:** DNA gene results.

Case ID	Gene	Mutation Classification	Chromosome	Function	Gene Region
1	*RET*	Missense	10	Normal	Exonic
2	*SLX4*	Missense	16	Loss	Exonic
3	*SDHA*	Missense	5	Normal	Exonic
4	*BCORL1*	Missense	X	Normal	Exonic
5	*ESR1*	Missense	6	Loss	Promoter; Exonic

**Table 3:** DNA results.

Case ID	Transcript ID	Transcript Variant	Protein Variant	Allele Frequency (%)	Variation	Mean Cov. TSO500
1	NM_020975.6; NM_001355216.1; ENST00000340058.6; NM_020630.6	c.1241G>A; c.479G>A	p.R414K; p.R160K	0.86	SNV	576
2	NM_032444.4	c.947A>C	p.N316T	4.73	SNV	624
3	NM_004168.4; ENST00000510361.5; NM_001294332.2; ENST00000264932.11; NM_001330758.2	c.1216G>A; c.1360G>A	p.A406T; p.A454T	0.95	SNV	642
4	NM_001184772.3; NM_001379451.1; NM_021946.5; NM_001379450.1; ENST00000540052.6	c.2869C>T	p.L957F	1.09	SNV	611
5	ENST00000206249.8; NM_001385568.1; NM_001291241.2; NM_001291230.2; NM_001385570.1; NM_001122741.2; NM_000125.4; NM_001385569.1; NM_001385571.1; NM_001122740.2; NST00000427531.6; NM_001328100.2; NM_001385572.1; ENST00000406599.5; M_001122742.2; ENST00000456483.3	c.166G>A; c.-1261G>A	p.E56K	1.03	SNV	743

## Data Availability

Direct data regarding DNA results are not allowed to be published due to the General Data Protection Regulation.
